# Incidence of Tumour Lysis Syndrome in Patients with Acute Myeloid Leukemia During Initiation of Therapy with Azacitidine and Venetoclax: A Retrospective Chart Review from a Canadian Single-Centre Perspective

**DOI:** 10.3390/curroncol32040213

**Published:** 2025-04-02

**Authors:** Tana Saiyin, Grace Christou, Mitchell Sabloff, Tina Crosbie, Kim-My Nguyen-Tham, Jill Fulcher

**Affiliations:** 1 Department of Medicine, The Ottawa Hospital, University of Ottawa, 501 Smyth Road, Ottawa, ON K1H 8L6, Canada; tsaiyin@toh.ca; 2 The Ottawa Hospital Leukemia Program, Department of Medicine, Division of Hematology, Ottawa Hospital Research Institute, University of Ottawa, 501 Smyth Road, Ottawa, ON K1H 8L6, Canadamsabloff@toh.ca (M.S.); 3 Department of Pharmacy, The Ottawa Hospital, 501 Smyth Road, Ottawa, ON K1H 8L6, Canada; tcrosbie@toh.ca (T.C.); kimmnguyen@toh.ca (K.-M.N.-T.)

**Keywords:** tumour lysis syndrome, acute myeloid leukemia, azacitidine, venetoclax

## Abstract

Azacitidine and venetoclax (Aza-Ven) are part of a new standard of care for elderly patients with Acute Myeloid Leukemia (AML) [In line with recommendations, patients with AML at our centre were routinely admitted during initiation of Aza-Ven for close monitoring for tumour lysis syndrome (TLS). However, hospitalization impacts patient experience and is a significant resource burden. The objectives of this study were to evaluate the incidence of TLS in this population and identify patients who could safely initiate therapy in our outpatient facility. Of the 48 patients who commenced Aza-Ven as inpatients, the incidence of TLS was 25% using Cairo–Bishop (CB) diagnostic criteria but was mostly due to transient increases in uric acid, phosphate or potassium that remained within the normal laboratory reference range. Using Howard diagnostic criteria, TLS incidence was only 2%. Patients who developed CB TLS had a significantly higher baseline white blood count (WBC; *p* = 0.01). Patients with WBC of less than 30 × 10^9^/L subsequently completed outpatient initiation of Aza-Ven (*n* = 15). Only one of these patients developed mild, transient TLS by CB criteria but not by Howard criteria. Our results demonstrate that a significant portion of patients could safely initiate Aza-Ven in our outpatient facility and avoid unnecessary hospitalization.

## 1. Introduction

Induction with intensive chemotherapy remains the standard-of-care therapy with curative intent for patients with Acute Myeloid Leukemia (AML) who are considered fit enough to tolerate the toxicity associated with this regimen [1,2]. In older or less fit patients, death during induction is significantly increased, with a therapy-related mortality rate greater than 50% for patients aged 80 and older [3]. As the median age at diagnosis of AML is 68 years [4], a significant proportion of these patients would be considered medically unfit for intensive chemotherapy due to advanced age, poor performance status, and/or baseline comorbidities [5]. The phase 3 landmark clinical trial VIALE-A [2] demonstrated that, for patients with AML who are elderly or unfit for intensive chemotherapy, the less intensive combination of azacitidine with venetoclax (Aza-Ven) could achieve a composite complete remission rate of 66.4%; red-cell- and platelet-transfusion-independence in 59.8% and 68.5% of patients, respectively; and a median overall survival of 14.7 months. Aza-Ven has subsequently been established as a new standard-of-care therapy in this patient population [1] and is approved in Canada for patients who are 75 years or older or medically unfit for intensive chemotherapy [6].

Venetoclax is a potent inhibitor of the B-cell lymphoma (BCL-2) protein, a key regulator of mitochondrial apoptotic signaling responsible for maintaining survival of AML blasts [7]. The efficacy of venetoclax-containing therapy has been associated with increased incidence of tumour lysis syndrome (TLS), which was initially observed in patients with Chronic Lymphocytic Leukemia [8,9]. TLS can result in life-threatening biochemical abnormalities that increase the risk of cardiac arrythmias, seizure, and acute kidney injury [10,11]. To mitigate the risk of TLS during Aza-Ven therapy in patients with AML, the European LeukemiaNet (ELN) recommendations include increased hydration, the prophylactic use of urid acid-lowering drugs, and cytoreduction of the white cell count (WBC) to <25 × 10^9^/L prior to therapy initiation [1]. In addition, a dose-escalation regimen is recommended for initiation of venetoclax with close monitoring for TLS [1]. To optimize the prevention and prompt management of TLS in the VIALE-A clinical trial, all patients were hospitalized before initiation of Aza-Ven therapy and until 24 h after reaching the maximum dose of venetoclax [2]. In line with this and with Cancer Care Ontario (CCO)guidance [12], it has been routine practice at The Ottawa Hospital to admit all patients for the first 4 days of Aza-Ven therapy for AML. This has imposed a significant burden on already over-stretched hospital resources and has a negative impact on patient experience.

The objectives of this study were to achieve the following:

1. Assess the incidence of TLS in our population of AML patients receiving inpatient Aza-Ven ramp-up.
2. Identify potential risk factors for TLS to facilitate the selection of patients at low risk of TLS who could safely start Aza-Ven therapy in the outpatient setting and be spared hospitalization.
3. Quantify the incidence of TLS in patients who subsequently received outpatient ramp-up of Aza-Ven therapy.

## 2. Materials and Methods

### 2.1. Study Design and Patient Characteristics

This was a retrospective health records review of a cohort of adult patients with AML who initiated Aza-Ven therapy at The Ottawa Hospital (TOH) between January 2022 and August 2024. Patients were identified using TOH Leukemia Program database and pharmacy records. The project was approved by the Ottawa Health Science Network Research Ethics Board.

In line with ELN [2] and CCO recommendations [12], all patients received standard TLS prophylaxis with intravenous fluids and a uricosuric agent (allopurinol), and those patients with a high WBC received cytoreduction with hydroxyurea, with the aim of reducing to a WBC less than 25 × 10^9^/L prior to starting Aza-Ven therapy. Venetoclax was administered over a three-day dose ramp-up schedule (50 mg on day 1, 100 mg on day 2, 200 mg on day 3, and onwards), which is a 50% dose reduction to account for coadministration with fluconazole, the standard-of-care fungal prophylaxis in this patient population at TOH. For patients who were admitted for inpatient ramp-up, blood chemistry for TLS monitoring was performed every 8 h after each venetoclax dose increase and for 24 h after the maximum dose was administered. Patients with no laboratory evidence of TLS and no other clinical complications were typically discharged on day 4 to finish the cycle as an outpatient and continue TLS bloodwork monitoring for up to 7 days after initiation of Aza-Ven therapy and twice weekly thereafter for the remainder of cycle 1.

Based on our initial study findings, patients with a baseline WBC of less than 30 x 10^9^/L without prior cytoreduction were identified as being at low risk of TLS. If these patients had an ECOG of 0–2 with the capacity to understand; if they adhered to the educational instructions regarding oral fluid intake, timing of venetoclax administration, and attendance at outpatient appointments; and if there were no concerns regarding tolerance of increased fluid intake, they were subsequently selected to receive Aza-Ven ramp-up as outpatients in the Medical Day-Care Unit (MDCU), an ambulatory hematology chemotherapy unit. These patients were scheduled for daily outpatient visits in MDCU for the first 7 days of Aza-Ven therapy. In advance of initiating therapy, all patients were started on allopurinol and educated by the pharmacists to increase their oral fluid intake to greater than 2L daily. They were instructed to take their first dose of venetoclax at home at 08.00 h on day 1 of their first cycle of therapy. Patients were booked appointments in MDCU for TLS bloodwork 6 to 8 h after taking the starting dose of venetoclax (50 mg), and they received a 1L intravenous bolus of saline while waiting for TLS bloodwork results. If there was no biochemical TLS, patients were administered their dose of azacitidine and approved to take the next dose increase of venetoclax (100 mg) at home at 08.00 h the following day. This process was repeated daily until 24 h after the maximum dose of venetoclax (200 mg) was administered. TLS bloodwork was repeated daily until day 7 of Aza-Ven and then twice weekly for the first 28-day cycle.

### 2.2. Identification of TLS

TLS was diagnosed using the Cairo–Bishop (CB) criteria [10], as well as the more recently revised criteria by Howard et al. [11]. Laboratory TLS by CB criteria is defined by two or more of potassium ≥6 mmol/L or ≥25% increase from baseline, hypocalcemia ≤1.75 mmol/L or ≥25% decrease from baseline, hyperphosphatemia ≥1.45 mmol/L or ≥25% increase from baseline, and hyperuricemia ≥476 mmol/L or ≥25% increase from baseline within three days before or seven days after therapy initiation [10]. The Howard criteria revised the CB criteria by excluding 25% change from baseline and specified that two or more abnormal laboratory values should be present within the same 24-h period [11]. Patients with laboratory TLS were considered to have clinical TLS by both criteria if there was documented cardiac arrythmia, seizure, or acute kidney injury defined as serum creatinine 1.5 times upper limit of normal (ULN) or 50% increase from baseline.

### 2.3. Statistical Analysis

Differences in baseline characteristics between patients developing TLS and those that did not develop TLS were assessed using two-tailed Mann–Whitney U test for continuous variables and Fischer’s exact test for categorical variables. A *p*-value < 0.05 was considered statistically significant.

## 3. Results

### 3.1. Patient and Disease Characteristics

A total of 66 patients with AML who received Aza-Ven therapy between January 2022 and August 2024 at TOH were included in our analysis. Forty-eight of these were hospitalized to receive Aza-Ven ramp-up as inpatients, and subsequently, eighteen patients started Aza-Ven therapy as outpatients.

### 3.2. Incidence of TLS in Patients Who Received Aza-Ven Ramp-Up as Inpatients

For the 48 patients that received inpatient Aza-Ven ramp-up, the distribution of male and female patients was 54% and 46%, respectively, with a median age of 72 (Table 1). Of these, 31 patients (65%) had newly diagnosed de novo AML, and 17 patients (35%) had either secondary or relapsed/refractory AML.

Twelve patients (25%) had an initial WBC greater than 25 × 10^9^/L and received hydroxyurea for cytoreduction prior to initiation of Aza-Ven. Post-cytoreduction, the median WBC on day 1 of Aza-Ven therapy was 5.25 × 10^9^/L. Although the aim of cytoreduction was to decrease the initial WBC to less than 25 × 10^9^/L before starting Aza-Ven, six patients who responded to cytoreduction with hydroxyurea had not yet met the target WBC at the time of initiating Aza-Ven (range of 27.7–68.4 × 10^9^/L). The median bone marrow blast percentage prior to starting Aza-Ven therapy for the 48 patients was 30%, and 21 of these (43.7%) had a bone marrow blast burden of greater than 50%. Two patients included in this study had persistent disease post-intensive induction chemotherapy, with less than 20% blasts at time of initiating Aza-Ven therapy.

In all, 37 of the 48 patients (77%) had at least one chronic comorbidity that potentially increased their risk of developing renal impairment from TLS: 6 patients (12%) had chronic kidney disease, 8 patients (17%) had diabetes mellitus, and 22 patients (46%) had hypertension. In total, 10 of the 48 patients (21%) had more than one of these comorbidities.

**Figure 1:**
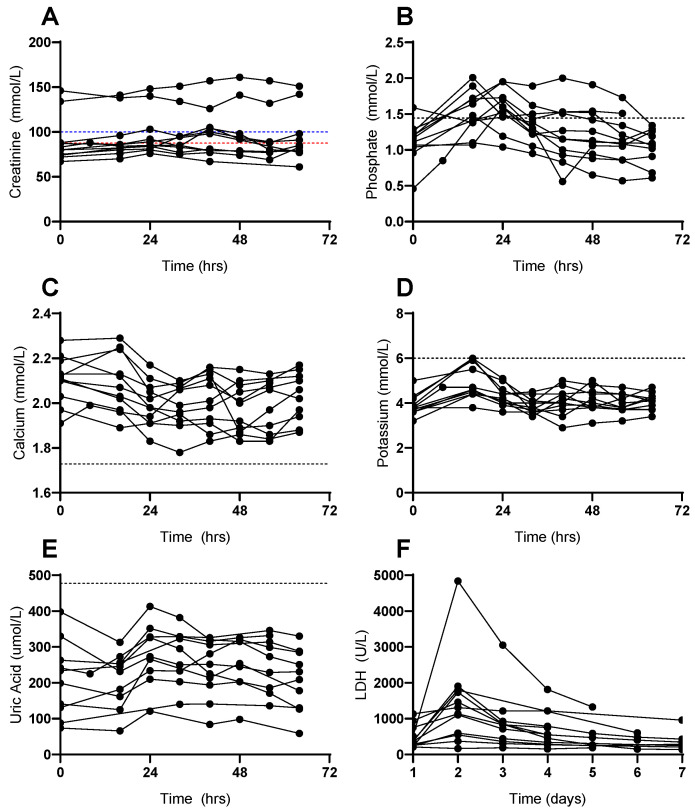
Patients with Acute Myeloid Leukemia (AML) who fulfilled the criteria for tumour lysis syndrome during inpatient azacitidine and venetoclax therapy: change in serum levels of (**A**) creatinine, (**B**) phosphate, (**C**) calcium, (**D**) potassium, (**E**) uric acid, and (**F**) lactose dehydrogenase (LDH) over initial 72 h of therapy. Dotted lines represent absolute laboratory values for the diagnosis of TLS, as defined by Cairo–Bishop and Howard criteria. (**A**) Red and blue lines represent upper limit of normal serum creatinine for female and male patients, respectively.

Twelve of these forty-eight patients (25%) met CB criteria for laboratory TLS, and of these, two patients (4%) met criteria for CB clinical TLS due to an increase in creatinine (Figure 1A). Seven of the twelve patients who met the diagnostic criteria of CB laboratory TLS did so due to 25% increases in uric acid and phosphate, but the phosphate increases did not reach > 2 mmol/L, and the absolute uric acid levels remained within the normal laboratory reference range (Figure 1B,C, respectively). Five of the twelve patients met CB criteria for TLS due to 25% increases in potassium and phosphate, but only two of these patients had potassium levels outside of the normal laboratory reference range, with a highest serum potassium level of 6 mmol/L. Both patients had a single elevated potassium value of 6 mmol/L in the first 24 h that was transient and had normalized upon the subsequent blood test, without intervention.

Baseline WBC before cytoreduction was significantly higher in patients who developed TLS by CB criteria compared to those patients who did not develop TLS (13.6 and 4.8 × 10^9^/L, respectively; *p* = 0.01) (Table 1). Twelve patients required cytoreduction, and post-cytoreduction, the WBC was also significantly higher in patients who developed CB laboratory TLS (9.9 vs. 4.6 × 10^9^/L, *p* = 0.04). Two of six patients who did not achieve a WBC of less than 25 × 10^9^/L prior to initiating Aza-Ven therapy developed TLS by CB laboratory criteria.

The median baseline bone marrow blast % was higher in those patients who developed TLS by CB criteria compared to those patients who did not (Table 1), but the difference did not quite reach statistical significance (65% vs. 30%, *p* = 0.06). There was no significant difference in baseline LDH levels in those patients who did and those who did not develop CB TLS. However, the two patients who developed clinical CB TLS had high initial LDH values of 758 and 1135 U/L (ULN reference range is 186 U/L). One of these patients also had chronic renal impairment, with a baseline creatinine of 212 μmol/L.

Those patients who developed TLS by CB criteria did not have higher baseline creatinine, potassium, phosphate, or uric acid levels compared to those patients who did develop CB TLS. There was no significant difference between the incidence of chronic renal failure, hypertension, and diabetes in patients who developed TLS compared to those who did not develop TLS (Table 1).

**Table 1:** Baseline characteristics for patients receiving azacitidine–venetoclax as inpatients (*n* = 48).

Characteristic	With Lab or Clinical TLS (*n* = 12)	Without TLS (*n* = 36)	*p* Value (Two-Tailed)
Age (years)	74.5 (55–85)	70 (43–84)	0.09
Male	7 (58)	19 (53)	0.99
**AML Type**			
De novo (%)	10 (83)	21 (58)	0.17
Secondary (%)	1 (8)	7 (19)	0.66
Therapy-related (%)	1 (8)	8 (22)	0.42
**Disease Burden**			
Baseline WBC (×10^9^/L)	13.6 (3.9–203.6)	4.8 (0.8–195.8)	0.01
WBC on day 1 of therapy	9.9 (1.9–38.4)	4.6 (0.3–68.4)	0.04
Bone marrow blasts (%)	65 (21–95)	30 (10–95)	0.06
Baseline LDH (mmol/L)	368.5 (210–1135)	263 (105–2325)	0.13
**Baseline TLS Parameters**			
Potassium (mmol/L)	3.7 (3.1–5)	4.3 (3.3–5.1)	0.01
Phosphate (mmol/L)	1.16 (0.96–1.89)	1.21 (0.68–2.07)	0.98
Calcium (mmol/L)	2.1 (1.91–2.29)	2.21 (1.86–2.48)	0.02
Uric acid (μmol/L)	216 (74–398)	240 (61–510)	0.35
Creatinine (μmol/L)	80 (41–212)	76 (37–367)	0.77
**Comorbidities**			
Diabetes mellitus (%)	3 (25)	5 (14)	0.39
Chronic kidney disease (%)	1 (8)	5 (14)	0.99
Hypertension (%)	6 (50)	16 (44)	0.75

Data are presented as median (range) or N (%) as appropriate. Abbreviations: TLS, tumour lysis syndrome; AML, acute myeloid leukemia; WBC; white cell count; LDH, lactate dehydrogenase. Normal range: WBC 3.5–10.5 × 10^9^/L; LDH 99–186 U/L; potassium 3.5–5.1 mmol/L; phosphate 0.81–1.40 mmol/L; calcium 2.24–2.58 mmol/L; uric acid 155–400 μmol/L; creatinine 49–84 μmol/L (female) or 62–100 μmol/L (male).

In 11 of the 12 patients who met criteria for CB laboratory TLS, this occurred within the first 48 h of initiating Aza-Ven, and 1 patient fulfilled the criteria for clinical TLS on day 6, with 25% increase in serum potassium (5.6 mmol/L) and phosphate (1.25 mmol/L), in addition to an increase in creatinine (116 μmmol/L from a baseline of 72 μmol/L). However, sample hemolysis, together with being on perindopril and spironolactone, may have contributed to the elevations in potassium. Only one patient who had high LDH (1310 U/L) was given rasburicase for TLS within 48 h of receiving Aza-Ven. All incidences of TLS were transient and resolved completely back to baseline within 7 days of monitoring.

Using Howard criteria for diagnosing TLS, only two patients (4%) met the criteria for TLS: one patient met laboratory TLS criteria, and the second fulfilled the criteria for laboratory and clinical TLS due to an increase in creatinine.

### 3.3. Incidence of TLS in Patients Who Received Aza-Ven Ramp-Up as Outpatients

Eighteen patients were subsequently selected to receive initiation of Aza-Ven therapy using the same venetoclax dose ramp-up but in the MDCU outpatient setting. Of these patients, three patients required admission for febrile neutropenia during the first week, thus interrupting further therapy. The remaining fifteen patients successfully completed their first cycle of Aza-Ven as outpatients (Table 2). The median age of these patients was 73 years (range 50–79). The median baseline WBC was 4.7 × 10^9^/L (0.9–26 × 10^9^/L), and the median bone marrow blast burden was 30%, with only two patients having BM blasts greater than 50%. Three of these fifteen patients had received prior intensive therapy for AML and had either refractory or relapsed disease with bone marrow blasts less than 20% at initiation of Aza-Ven therapy. None of the 15 patients who completed outpatient therapy developed clinical TLS or required targeted treatment for TLS. Only one patient met CB diagnostic criteria for laboratory TLS: this was on day 4 of therapy and due to an absolute phosphate >1.45 mmol/L and a 25% increase in uric acid that did not reach the absolute diagnostic level of 476 μmol/L (increased from 324 μmol/L to 431 μmol/L). This patient had an elevated baseline phosphate (1.51 mmol/L), which peaked on day 2 at 1.61 mmol/L). The patient was monitored for 7 days but did not develop clinically significant TLS and did not require additional treatment or hospital admission.

### 3.4. Patient Outcomes

In our cohort of patients with AML who received Aza-Ven therapy, composite remission rate (complete remission + complete remission with incomplete count recovery) was 65% after cycle 1 and 78% after cycle 2. Two of the sixty-six patients in our cohort died within 30 days of therapy initiation, neither of whom had received outpatient initiation of Aza-Ven therapy or had developed TLS.

## 4. Discussion

The combination of the BCL-2 inhibitor, venetoclax, and the hypomethylating agent (HMA) azacitidine is now considered a new standard of care for the treatment of elderly and/or unfit patients with AML [1]. In the landmark VIALE-A clinical trial, all patients were hospitalized for pre-hydration and close monitoring for the first 4 days of a dose-escalation regimen [2]. Although many centres, including The Ottawa Hospital (TOH), have mirrored this practice, hospitalization increases the cost of therapy, imposes considerable burden on hospital resources, and has a negative impact on patient experience. The results of the study presented here add to an increasing body of data from real-world studies confirming that hospital admission is not necessary for all patients with AML during initiation of this therapy [13,14,15].

The reported incidence of laboratory TLS during venetoclax-containing therapy in patients with AML varies from 1.1% in the VIALE-A clinical trial [2] to as high as 49.9% in a subset of patients in a real-life study at the University of Texas MD Anderson Cancer Center [14]. This variability in incidence can be explained by the different criteria used to diagnose TLS. Studies that used the Cairo–Bishop (CB) diagnostic criteria report a higher incidence of laboratory TLS compared to studies that used the Howard criteria. There has been recent debate about which diagnostic criteria for TLS is more clinically relevant. Using CB criteria, Diao and colleagues [14] reported laboratory TLS in 59 of 148 (39.9%) patients with AML treated with venetoclax-based therapy, but only 5.4% of these patients had laboratory values higher than the normal laboratory reference range, and only 2.7% met the CB criteria for clinical TLS. Most of these cases (76.3%) were caused by elevations in serum uric acid and/or phosphate. Arora and coworkers [13] demonstrated laboratory TLS using CB criteria in 19 out of 106 patients (17.9%), but only 2 of these 19 patients had TLS parameters that exceeded the normal laboratory reference ranges. The other 17 patients had biochemical values that remained within the normal range but fulfilled the CB diagnostic criteria due to increases that were ≥25% from baseline. In our study, 25% of AML patients treated with Aza-Ven fulfilled CB diagnostic criteria for laboratory TLS, but these were mostly due to increases in phosphate and uric acid that either remained within the normal reference range or were only slightly above the upper level of normal. None of these patients had changes in potassium, calcium, or uric acid that reached the absolute thresholds for CB laboratory TLS of 6 mmol/L, 1.75 mmol/L, or 476 μmol/L, respectively.

The clinical relevance of increases in TLS parameters that are not outside the normal laboratory reference range was questioned by Howard and colleagues, and in their revision of the diagnostic criteria for TLS, increases in potassium, phosphate, and uric acid of 25% from baseline are only considered to be of clinical value when the baseline value is higher than normal [11]. Studies that used the Howard TLS diagnostic criteria report a much lower incidence of TLS in patients with AML during venetoclax-containing therapy. In the VIALE-A clinical trial, only 3 out of 283 patients (1.1%) with AML who were treated with Aza-Ven met laboratory TLS by Howard criteria, and all of these were due to transient biochemical changes [2]. Similarly, Rowe and coworkers reported a 4% incidence of laboratory TLS using Howard diagnostic criteria in a study of 100 patients with AML treated with venetoclax-containing therapy [15], and Pelcovits et al. reported only one episode of laboratory TLS in a cohort of 41 patients with AML treated with venetoclax–HMA therapy [16]. Arora and coworkers reported the incidence of laboratory TLS using both CB and Howard criteria in 106 patients with AML who received venetoclax with either azacitidine or decitabine [13]. Using CB criteria, 18% of patients fulfilled the diagnosis of laboratory TLS, and 5% had clinical TLS. However, only 2% of patients had increases in TLS parameters that fulfilled the Howard diagnostic criteria for laboratory TLS. We confirmed a similar discrepancy in our patient population depending on which TLS diagnostic criteria was used—the incidence of laboratory TLS was 25% using CB criteria and only 2% using Howard criteria. All increases in TLS biochemical parameters observed in our study were minor and transient, resolving within the 7 days of monitoring. Observations from the study reported here and by others [2,13,15,16] suggest that the Howard diagnostic criteria may provide a more clinically relevant assessment of TLS in the context of patients with AML treated with venetoclax–HMA therapy. However, further studies are needed to confirm which diagnostic criteria should be used to monitor for TLS in this population of patients.

Differences in the CB and Howard diagnostic criteria have been highlighted recently by a panel of TLS experts that included Drs. Cairo, Bishop, and Howard [17]. This panel acknowledged the need to update TLS guidelines to reflect the increased risk posed by the newer, potent, targeted anticancer agents and immunotherapies. Using a multi-stage, systematic process, they evaluated current clinical practice and developed consensus guidelines that focused on TLS risk assessment, prophylaxis, and management [17]. The authors acknowledged that the heterogeneity in the risk of developing TLS depends not only on tumour type and bulk but also on specific patient characteristics in particular baseline/pre-treatment hydration status and renal function. Aggressive hydration is emphasized as the cornerstone of TLS prophylaxis and management, as it allows for dilution of potentially harmful levels of solutes from tumour lysis. AML with a WBC of less than 25 × 10^9^/L is classified by the authors as intermediate risk of developing TLS. The guidelines recommend a daily oral fluid intake of 2–3 L/m^2^ prior to therapy initiation and until the end of the day following therapy completion, and they advise supplementing this with additional intravenous fluids for patients at intermediate risk of TLS [17]. Although venetoclax is classified by the panel as a chemotherapeutic agent with a high risk of TLS, the authors acknowledge that tumour debulking prior to initiation of therapy and the use of a dose-escalation strategy can mitigate the risk of TLS in venetoclax-containing therapies. We adhered to these recommendations in the study presented here and demonstrated a very low incidence of transient TLS that did not have any clinically relevant impact. The crucial role of adequate hydration in this clinical setting is demonstrated by Diao and colleagues [14], who reported the highest incidence of TLS of 44.9% in those patients who received less than 500 mL of intravenous fluids daily during Aza-Ven therapy. Incidence was much less in those patients who received at least 1.5L of intravenous fluids per day.

With patient education and careful optimization of pre-treatment hydration status, initiation of allopurinol in advance of therapy and administration of additional intravenous hydration during the initial 3-4 days of venetoclax ramp-up, patients with a baseline WBC less than 30 × 10^9^/L could safely receive Aza-Ven in the outpatient setting. Other indices of tumour proliferation, such as bone marrow blast volume and elevated baseline LDH, are additional indicators of increased risk of TLS. Diao and colleagues demonstrated that a one natural-log unit increase in LDH resulted in a 1.8-fold increased likelihood of developing laboratory TLS by CB criteria in 148 elderly patients with AML who received venetoclax-based low-intensity therapy. In our study, the 2 patients who developed clinical TLS using CB criteria had LDH levels 4-6-times ULN. Patient-specific factors, in particular, ability to tolerate an adequate hydration regimen and baseline renal function, should also be taken into consideration when assessing risk of TLS and selecting patients for outpatient initiation of Aza-Ven therapy.

We acknowledge that our study has significant limitations. It is a single-centre retrospective study, and the total number of patients is small. However, the data are reassuring in confirming a low incidence of clinically significant TLS in our patient population and that outpatient initiation of Aza-Ven therapy is possible and safe in selected patients using the regimen we describe. Only one patient met the criteria for CB laboratory TLS and did not require any additional intervention. Our outpatient protocol enables close monitoring for TLS and, if needed, adjustment in therapy and prompt hospital admission for further management.

The incidence of TLS and disease remission rates for our cohort of patients with AML who received Aza-Ven in both the inpatient and outpatient settings are in line with those reported in the landmark VIALE-A clinical trial [2]. Our study adds to that of Pelcovitis and colleagues [16], who successfully treated as outpatients 41 patients with AML with HMA/venetoclax who had low-volume disease.

## 5. Conclusions

With careful patient selection and education, adherence to a specified hydration regimen, and close TLS monitoring during the initial week of therapy, a significant proportion of patients with AML could be start=ed on Aza-Ven therapy in the outpatient setting and thereby improve overall patient experience, decrease the cost of therapy, and relieve pressure on hospital resources.

## Figures and Tables

**Table 2:** Baseline characteristics for patients receiving azacitidine–venetoclax as outpatients (*n* = 15).

**Age (years)**	73 (50–79)
**Male**	11 (71)
**AML Type**	
De novo (%)	12 (80)
Secondary (%)	2 (13)
Therapy-related (%)	1 (7)
**Disease Burden**	
Baseline WBC (×10^9^/L)	4.7 (0.9–26)
Bone marrow blasts (%)	30 (8–80)
Baseline LDH (U/L)	222 (138–684)
**Baseline TLS Parameters**	
Potassium (mmol/L)	4.3 (3.8–5.1)
Phosphate (mmol/L)	1.31 (0.87–1.84)
Calcium (mmol/L)	2.27 (2.09–2.59)
Uric acid (μmol/L)	278 (126–425)
Creatinine (μmol/L)	83 (57–265)
**Comorbidities**	
Diabetes mellitus (%)	2 (13)
Chronic kidney disease (%)	1 (7)
Hypertension (%)	4 (27)

Data are presented as median (range) or N (%) as appropriate. Abbreviations: TLS, tumour lysis syndrome; AML, acute myeloid leukemia; WBC; white cell count; LDH, lactate dehydrogenase. Normal range: WBC, 3.5–10.5 × 10^9^/L; LDH, 99–186 U/L; potassium, 3.5–5.1 mmol/L; phosphate, 0.81–1.40 mmol/L; calcium, 2.24–2.58 mmol/L; uric acid, 155–400 μmol/L; creatinine, 49–84 μmol/L (female) or 62–100 μmol/L (male).

## Data Availability

The original contributions presented in this study are included in the article. Further inquiries can be directed to the corresponding author.

## Abbreviations

The following abbreviations are used in this manuscript:

MDPI	Multidisciplinary Digital Publishing Institute
DOAJ	Directory of Open Access Journals
TLS	tumour lysis syndrome
AML	acute myeloid leukemia
WBC	white cell count
Aza-Ven	azacitidine–venetoclax
CB	Cairo–Bishop
ELN	European LeukemiaNet
LDH	lactate dehydrogenase
TOH	The Ottawa Hospital
ULN	upper limit of normal
CCO	Cancer Care Ontario
